# Muscle carbonic anhydrase III levels in normal and muscular dystrophia afflicted chickens

**DOI:** 10.1186/1751-0147-54-34

**Published:** 2012-05-29

**Authors:** Toshiho Nishita, Daisuke Yorifuji, Kensuke Orito, Nobutsune Ichihara, Kazuyoshi Arishima

**Affiliations:** 1 Laboratory of Veterinary Physiology 1, School of Veterinary Medicine, Azabu, University, 1-17-71 Fuchinobe, Sagamihara, Kanagawa, 252-5201, Japan; 2 Laboratory of Veterinary Physiology 2, School of Veterinary Medicine, Azabu, University, 1-17-71 Fuchinobe, Sagamihara, Kanagawa, 252-5201, Japan; 3 Veterinary Anatomy 1, School of Veterinary Medicine, Azabu University, 1-17-71, Fuchinobe, Sagamihara, Kanagawa, 229-8501, Japan; 4 Veterinary Anatomy 2, School of Veterinary Medicine, Azabu University, 1-17-71, Fuchinobe, Sagamihara, Kanagawa, 229-8501, Japan

**Keywords:** Chicken, carbonic anhydrase III, Muscular dystrophy, Avian disease, ELISA, Immunohistochemistry

## Abstract

**Background:**

The levels and immunohistochemical localization of muscle carbonic anhydrase III (CA-III) in healthy chickens and in muscular dystrophia affected (DA) chickens show that the muscles of diseased animal undergo a progressive increase of enzyme activity.

**Methods:**

An enzyme-linked immunoassay was used to assess the CA-III levels in the muscles and other tissues from eight normal White Leghorn chickens and in two chickens with muscular dystrophy. Immunohistochemical localization of the enzyme in the muscles of these animals was also determined.

**Results:**

The levels of CA-III in the tensor fasciae latae and the superficial pectoral muscles of the DA chickens were higher than the level in normal chickens. The concentrations of CA-III in erythrocytes and plasma from diseased chickens were approximately 15-fold and 1.4-fold higher than in the normal chickens, respectively. In the superficial pectoral and the tensor fasciae latae muscles of diseased chickens, the numbers of strongly stained and weakly stained fibers were greater than that in the normal chickens.

**Conclusion:**

The levels of CA-III in the superficial pectoral muscle, the tensor fasciae latae muscle, plasma and erythrocytes from the chickens with muscular dystrophy were higher than found in normal chickens.

## Background

Avian muscular dystrophy is inherited as a simple autosomal recessive defect [1]. In dystrophia affected (DA) chickens, the fast-twitch pectoralis superficialis muscle undergoes a progressive loss of function. Along with the muscular weakness, histopathological changes [2], ultrastructual abnormalities [3] and large elevations in certain serum enzymes that escape from the affected muscles occur [4]. These changes are similar to those found in human Duchenne dystrophy. Hoffman *et al.*[5] reported that dystrophin, a large membrane-associated cytoskeletal protein, was absent in the affected muscles of Duchenne dystrophy. However, dystrophin is present in both normal and DA (line 413) chicken muscle indicating that this protein cannot be implicated in the pathogenesis of avian muscle dystrophy [6]. Although the DA chicken has been established as an animal model of muscular dystrophy in humans, the mechanism leading to muscle cell degeneration remains unknown.

Mizuno [7] and Park *et al.*[8] reported that superoxide dismutase activity in the superficial pectoral muscle of DA chickens was significantly elevated. Chown *et al.*[9] reported that the carbonic anhydrase III (CA-III) level in the pectoral muscle of DA chickens was approximately three fold higher than in normal birds. CA-III may have a role in scavenging oxygen radicals and thereby protecting cells from oxidative damage [10,11]. However, the immunohistochemical localization of CA-III in the muscles of DA chicken has not been reported.

The aim of the present study was to determine the concentrations and localization of CA-III in normal and DA chickens.

## Methods

### Animals and tissue samples

Normal male White Leghorn (WL) chickens, (Line M) 59 weeks old (n = 5) (Nisseiken Co., Ltd., Tokyo, Japan) and normal female WL chickens (LOHMAN LSL-LITE) 25 weeks old (n = 3) (Isogaya Yokeien, Tochigi, Japan) were used. Two male chickens with muscular dystrophy (A and B: 16 weeks old; New Hampshire line 413) were provided by Nagoya University Avian Bioscience Research Center (Nagoya, Aichi, Japan).

All experiments were performed according to the guidelines of the Laboratory Animal Care Committee of Azabu University and complied with the Japanese Animal Welfare Guide. The animals were euthanized by an overdose of pentobarbital (Nembutal^®^, Dainippon Sumitomo Pharma, Osaka, Japan) at the end of the study.

### Sampling

Before euthanasia, lithium heparin treated blood samples were taken and centrifuged at 4500 × *g* for 15 min at 4°C. The erythrocytes obtained were lysed with an equal volume of distilled water and centrifuged at 27000 × *g* for 30 min at 4°C. Hemolysate and plasma samples were stored at −20°C until analyzed. Hemolysate samples (0.1 mL) diluted from 1:4,000 to 1:16,000 and plasma samples diluted from 1:5 to 1:10 in 50 mM Tris–HCl (pH 7.5) containing 0.3% bovine serum albumin (BSA), 0.9% NaCl, 0.01% thimerosal, and 10 mM EDTA (buffer A) were subjected to duplicate ELISA.

Samples of the superficial pectoral, the tensor fasciae latae and the obliquus externus abdominis muscles, and of the kidneys, liver, lung and cardiac muscles taken at necropsy and one gram of each of these tissues were homogenized with 1 volume of 0.01 M Tris–HCl (pH 8.0) and then centrifuged at 4°C for 30 min at 27000 × *g*. The soluble fractions were used for analysis. Also, samples of the three muscles were immediately fixed in neutralized 10% formalin and Bouin’s solution and later dehydrated with a graded series of alcohols, cleared with xylene and then embedded in paraffin wax blocks. Four *μ*m sections were used for immunohistochemistry.

### Hemoglobin assay

The hemoglobin concentrations in the hemolysates were measured by the sodium lauryl sulfate-hemoglobin method using a hemoglobin B test (Wako Pure Chemical Industries Ltd., Tokyo, Japan).

### Electrophoretic procedures and western blotting

Western blotting was performed as previously described [12]. Briefly, adequate volumes of the hemolysate and purified chicken CA-III were separated by using the PhastSystem (Pharmacia Biotech, Uppsala, Sweden) and transferred to Immobilon PVDF transfer membrane (Millipore Corp, Bedford, Mass, USA) by means of a commercially available transfer system. The buffer used in the electrophoretic transfer contained 25 mM Tris (pH 8.3), 192 mM glycine, 0.1% sodium dodecyl sulfate (SDS), and 15% methanol. CA-III was detected using rabbit antiserum to chicken CA-III previously produced in our laboratory [13]. This antiserum in 5 mL of buffer A was diluted to 1:2000. After incubation with the primary antibody, the membranes were washed in 0.15 M phosphate-buffered saline (PBS) containing 0.05% Tween (PBS-Tween) and then incubated with 1:4,000 peroxidase-conjugated goat anti-rabbit IgG (Kirkegaard & Perry Laboratories Inc., Gaithersburg, MD, USA) in 5 mL of buffer A. The membranes were washed again with PBS-Tween and then incubated for approximately 5 min in pH 7.6, 0.05 M Tris–HCl containing 0.02% H_2_O_2_ and 0.2 mM 3.3’ diaminobentidine-tetrahydrochloride (DAB – 4HCl).

### Determination of CA-III levels

The CA-III concentrations in several chicken tissues were determined using the previously described competitive ELISA method [13]. Briefly, a flat-bottom micro-ELISA plate (Maxisorp Nunc-Immuno Plate; Nunc. Roskilde, Denmark) was incubated for 16 h at 4°C in 0.1 mL of pH 9.6, 0.1 M NaHCO_3_ containing 0.03 mg/mL of the antibody to chicken CA-III. The plates were then washed 3 times with 0.3 mL of PBS and blocked at 23°C for 30 min with 0.2 mL of 0.5% BSA in 0.05 M Tris–HCl (pH 8.0). Each well was then washed 3 times with 0.3 mL of PBS-Tween. Duplicate ELISAs were performed using purified CA-III (6–800 ng/mL), biotinylated chicken CA-III and tissue samples were diluted with buffer A, respectively. Tissue extracts (0.1 mL) diluted 50–5,000 with buffer A were subjected to immunoassay in duplicate. The biotinylated CA-III was allowed to compete with the standard CA-III or the tissues samples and incubated for 16 h at 4°C. Each well was then washed 3 times with PBS-Tween, and 0.1 mL/well of avidin and biotinylated horseradish peroxidase complex (ABC reagent, Wako Pure Chemical Industries Ltd, Tokyo, Japan) was added. After 30 min, each well was washed 3 times with PBS-Tween. Peroxidase activity was measured after the addition of 0.1 mL of the ABTS microwell peroxidase substrate (Kirkegaard & Perry Laboratories Inc,). The reaction was terminated after 10 min by the addition 0.1 mL of 1% SDS, and the absorbance at 405 nm was recorded on an automatic ELISA (SH-1000; Corona Electric Co., Ltd., Ibaraki, Japan).

The concentrations of CA-II in the erythrocytes from the chickens with muscular dystrophy were determined by the competitive ELISA method as previously reported [14].

### Immunohistochemistry

Endogenous peroxidase activity was blocked in the deparaffinized, rehydrated tissue sections by using 0.3% H_2_O_2_ in methanol, followed by immersion in normal goat serum (2% in PBS) for 20 min to block the FC receptors. Monospecific antisera (diluted 1:2,000) against chicken CA-III localized to the respective isozymes in a 1-h–long primary reaction were used. Antibody binding was visualized using the Vectastain Elite avidin-biotin-peroxidase complex kit (ABC-POD reagent kit; Vector, Burlingame, CA., USA) and DAB – 4HCl according to the manufacturer’s protocol. Samples were observed and photographed under a light microscope; the cell count was determined by densitometry. The percentage of fiber type composition in the muscle samples of the normal and DA chickens was calculated from the immunohistochemical localization of CA-III.

## Results

### Western blotting analysis

**Figure 1:**
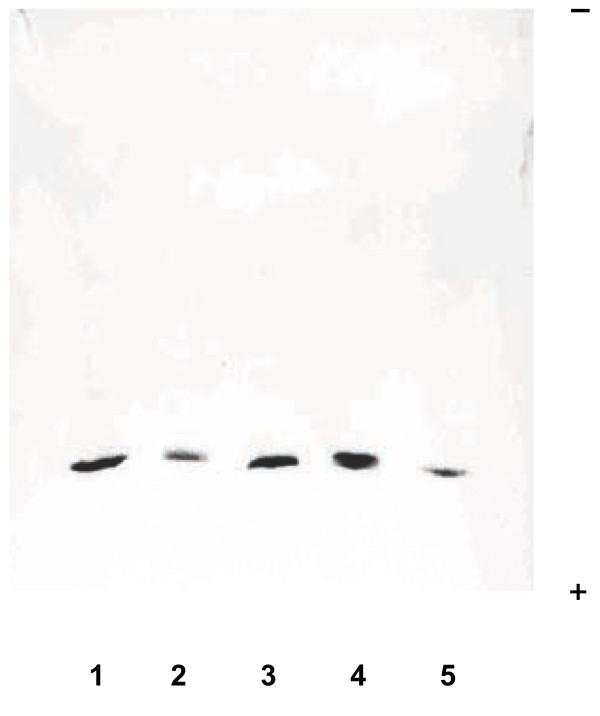
**Western blots of the muscle extracts from normal and dystrophic chickens separated by SDS-PAGE and analyzed using rabbit anti-chicken CA-III antiserum.** Lane 1, extract of the superficial pectoral muscle from a dystrophic chicken; lane 2, extract of the superficial pectoral muscle from a normal chicken; lane 3, extract of the tensor fasciae latae muscle from a normal chicken; lane 4, extract of the tensor fasciae latae muscle from a dystrophic chicken; and lane 5, purified chicken CA-III.

**Figure 2:**
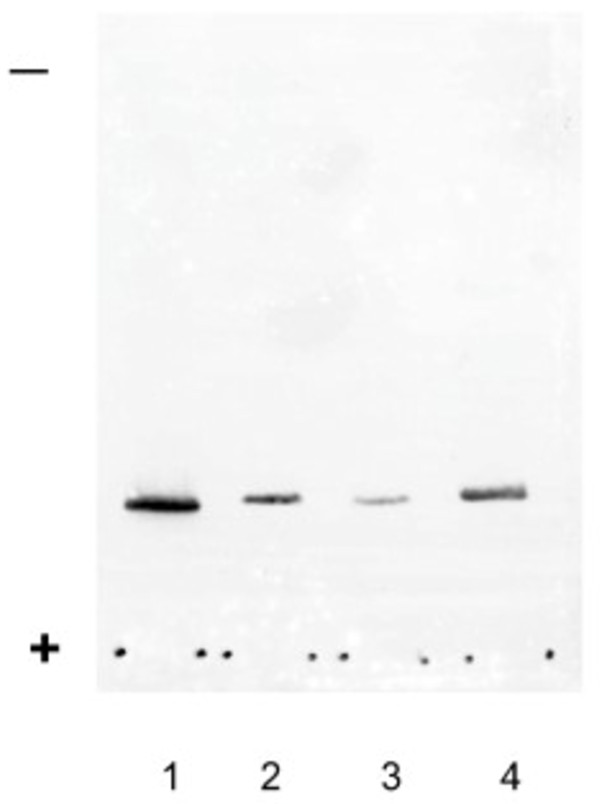
**Western blot of the hemolysate from normal and dystrophic chickens separated by SDS-PAGE.** Lane 1, purified chicken CA-III; lane 2, hemolysate from a dystrophic chicken; lane 3, hemolysate from a normal male chicken; and lane 4, hemolysate from a normal female chicken.

Figure 1 shows a single band on the western blot probed with anti-chicken CA-III serum from the extracts of the superficial pectoral and tensor fasciae latae muscles, and purified chicken CA-III. The molecular weight of CA-III in the extracts from the normal WL-chickens and those with muscular dystrophy were similar to that of the purified chicken CA-III. The western blot of Figure 2 shows that CA-III was present in the hemolysates of DA, female WL, male WL chickens. The molecular weight of these bands was approximately 28,000.

### CA-III and CA-II levels

The concentrations of CA-III in the tissue samples from normal and DA chickens determined by competitive ELISA assays are shown in Table 1. For the normal chickens, the highest content of CA-III was found in the obliquus externus abdominis muscle. The levels of CA-III in the obliquus externus abdominis muscle did not differ between normal and DA chickens. The levels of CA-III in the tensor fasciae latae muscle of the DA chickens was approximately 3-fold higher than that of normal animals and approximately 27-fold higher in the superficial pectoral muscle of the DA chickens. The CA-III levels in kidney and liver and in cardiac muscles were less than in skeletal muscles. The CA-III levels in the liver and lung, and in cardiac muscles of DA chickens were approximately 3–7 fold higher than in normal chickens. The levels of CA-III in the kidneys of the DA chickens were approximately one-third of that seen in the controls.

**Table 1:** Concentrations of carbonic anhydrase III in the organ samples from normal male and muscular dystrophic chickens

Samples	normal male chicken (n = 5)	male dystrophic chicken		dystrophy/normal
		A	B	
	(*μ*g/g of wet)	(*μ*g/g of wet)	(*μ*g/g of wet)	
Obliquus externus abdominis muscle	99 ± 49	81.8	62.2	0.6
Tensor fasciae latae muscle	61 ± 45	207.7	175.9	3.1
Superficial pectoral muscle	8.4 ± 7.8	274.9	173.8	26.6
Kidney	3.2 ± 3.8	1.4	1.1	0.4
Liver	0.2 ± 1.1	1.3	1.6	7.2
Lung	1.0 ± 0.4	5.3	4.2	4.8
Cardiac muscle	0.2 ± 0.1	0.6	0.6	3
	(*μ*g/g of Hb)	(*μ*g/g of Hb)	(*μ*g/g of Hb)	
Erythrocyte	1.9 ± 0.8^a)^	27.5	28.7	15
	(ng/mL)	(ng/mL)	(ng/mL)	
Plasma	105 ± 37^a)^	149	144	1.4

The levels of CA-III in the liver and the superficial pectoral muscle of normal female chickens were 0.9 ± 1.0 and 21 ± 5.3 *μ*g/g wet tissues, respectively, which were significantly higher than in normal males (*P* < 0.05). CA-III levels in other tissues did not differ between the sex of normal chickens.

The concentrations of CA-III in the erythrocytes of the chickens with muscular dystrophy were approximately 15-fold higher than those of the controls. Moreover, the concentrations of CA-III in plasma of DA chickens were approximately 1.4-fold higher than those in normal chickens. The concentrations of CA-II in the erythrocytes of the DA chickens No. A and B were 93.6 and 119.6 mg/g of Hb, respectively, and 113.6 ± 32.3 mg/g of Hb in erythrocytes of normal male chickens (17 weeks old) [14].

### Immunohistochemical staining of CA-III

**Figure 3:**
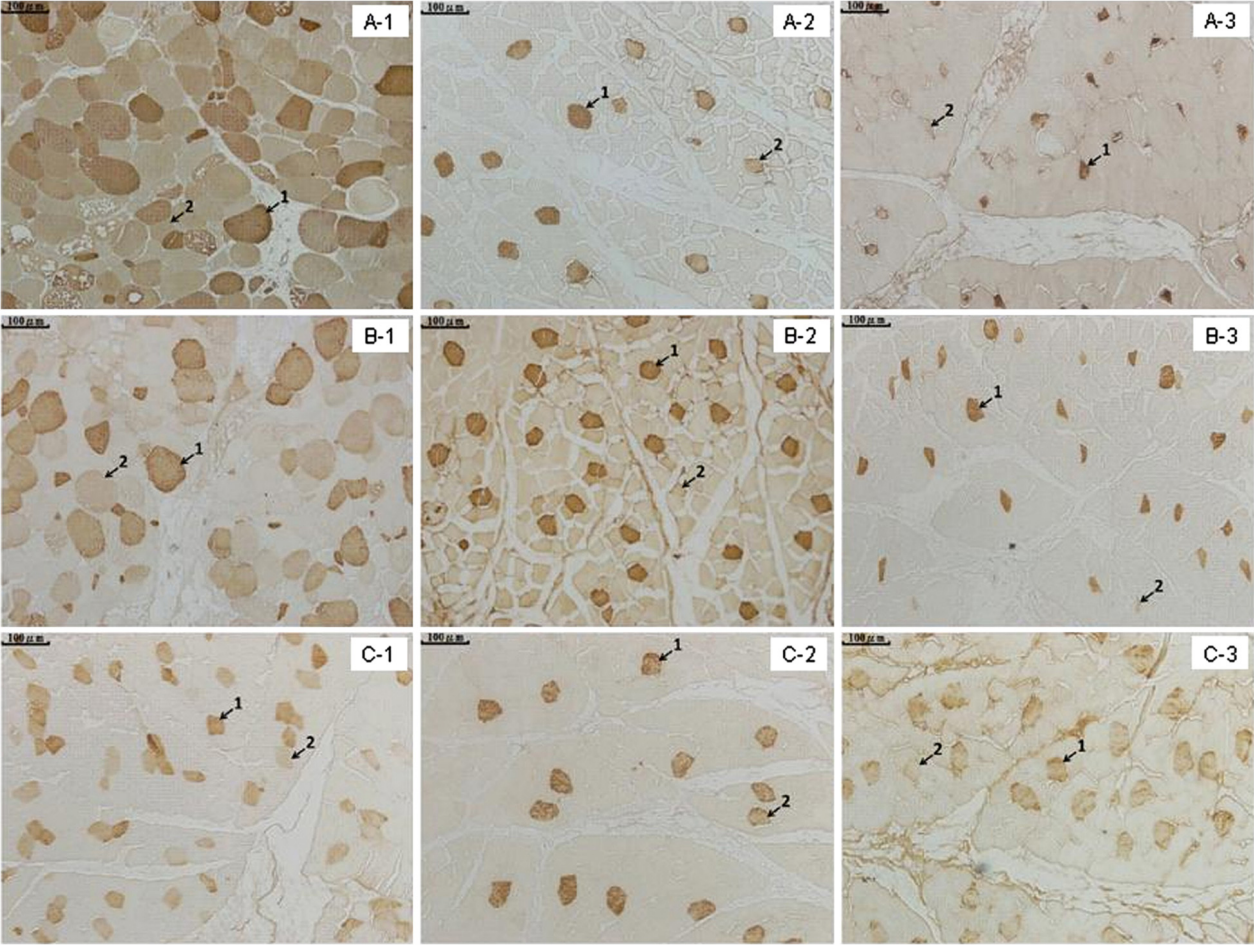
**Immunohistochemical localization of CA-III in the muscles from normal and muscular dystrophic chickens.A-1**, the superficial pectoral muscle of a dystrophic chicken A; **A-2**, the tensor fasciae latae muscle of a dystrophic chicken A; **A-3**, the obliquus externus abdominis muscle of a dystrophic chicken A; **B-1**, the superficial pectoral muscle of a dystrophic chicken B; **B-2**, the tensor fasciae latae muscle of a dystrophic chicken B; **B-3**, the obliquus externus abdominis muscle of a dystrophic chicken B; **C-1**, the superficial pectoral muscle of a normal chicken; **C-2**, the tensor fasciae latae muscle of a normal chicken; and **C-3**, the obliquus externus abdominis muscle of a normal chicken. Arrow 1 indicates strongly stained CA-III. Arrow 2 indicates weakly stained CA-III. Scale bars: 100 μm.

The immunohistochemical reaction of the CA-III antiserum observed in the muscle fibers of the normal and DA chickens are shown in Figure 3. The shape of these fibers in the CA-III-stained normal chicken muscle was a polygon (Figure 3 C-1–3), whereas they were hypertrophied and pathologically rounded in the fibers of the superficial pectoral muscle from DA chickens (Figure 3 A-1, B-1).

Vacuolar degeneration was also observed in the superficial pectoral muscles of the DA chickens, while such lesions were not observed in the muscle fibers from the tensor fasciae latae muscle and the obliquus externus abdominis muscle.

The muscle fibers were classified into 3 types depending on the immunohistochemical staining intensity of CA-III; strongly, weakly, and not stained. Table 2 shows the proportions of the 3 kinds of staining patterns in the muscles of normal and DA chickens. In the superficial pectoral muscles of the DA animals, the numbers of strongly stained fibers were about 12- and 9-fold greater than found in normal chickens, respectively; while the numbers of weakly stained fibers were about 3- and 2-times greater. In the tensor fasciae latae muscles of the DA chickens, the numbers of strongly stained fibers were about 2- and 4-times higher that of normal chickens, and the numbers of weakly stained fibers were about 2-and 4-times higher. In the obliquus externus abdominis muscle of the DA chickens, the numbers of strongly stained fibers were about 2- and 3-times greater than that of normal chickens. The numbers of weakly stained fibers were about 0.6- and 0.4-times less than that of normal chickens.

**Table 2:** The number and classification of the muscle fibers from normal and muscular dystrophic chickens based on the intensity of CA-III immunostaining

		Stain reactions of CA-III			
Muscle	Chicken	Strong	Weak	Negative	Total cell count
Superficial pectoral muscle	Dystrophy				
	A	246	253	501	1,000
	B	177	170	653	1,000
	Normal	20	78	902	1,000
Tensor fasciae latae muscle	Dystrophy				
	A	52	72	876	1,000
	B	104	183	713	1,000
	Normal	24	48	928	1,000
Obliquus externus abdominis muscle	Dystrophy				
	A	61	62	877	1,000
	B	103	35	862	1,000
	Normal	38	94	868	1,000

^a)^: Nishita *et al.*[10].

## Discussion

### CA-III levels in chicken tissue

The levels of CA-III in the liver of DA chickens were approximately 7-fold higher than that of normal chickens (males and females). In the lung and cardiac muscle of DA chickens, the levels of CA-III were 3–5- times higher than in normal chickens, but the kidney level of CA-III was approximately one-third of that present in normal individuals and vastly increased compared to other organs analyzed.

Chown *et al.*[9] reported that the concentration of CA-III in adult chicken pectoral muscle was 2–4 *μ*g/g wet tissue and 60–500 *μ*g/g wet tissue in the leg (red muscles). Mizuno [7] reported that Mn SOD activities in the superficial pectoral muscles of DA chickens declined with age as in normal chickens, whereas Cu-Zn SOD activities increased until 4 weeks of age. The activity of this enzyme was still twice as high as that of the normal birds at 16 weeks of age. In control chickens, both Cu-Zn SOD and Mn SOD activities declined with age. Increased activities of SOD during the early stages of development suggest an increased turnover of active oxygen species caused by early onset of avian muscular dystrophy. These biochemical changes are considered to represent altered metabolism within muscle cells and not to invasion by phagocytes or other cells.

Mizuno [7,15] also suggested that increased levels of catalase, glutathione peroxidase, and glutathione reductase in DA chickens indicates increased capacity for oxygen-free radical turnover within the muscle cells and that oxygen-free radicals and related activated oxygen species may play a role in inducing cellular damage.

Gailly *et al*. [16] reported that exposure of human kidney proximal tubule immortalized HK-2 cells, an established model for normal human PT cells, to 1 mM H_2_O_2_ induced a significant increase in CA-III mRNA expression. In the present study, the elevation of CA-III in the muscle fibers and other tissues from DA chickens is probably induced by an increase in the reactive oxygen species present. Comparatively, the obliquus externus abdominis muscles of normal and DA chickens, which contain high levels of CA-III, are probably not affected by the increased level of reactive oxygen species. Therefore, we propose that chicken CA-III is useful biomarker of radical-induced damage.

### Immunohistochemical staining of CA-III

Barnard *et al*. [17] showed 5 major fiber types in chicken skeletal muscles namely, I, IIA, IIB, IIIA, and IIIB. Fibers of types I, IIA, and IIB in the pectoral muscle of 50–80 day old normal chickens composed 0%, <1%, and >99% of the tissue. Although the localizations of CA-III into specific fiber types were not made in this study, about 2% of fibers in the superficial pectoral muscles of normal chickens reacted strongly and 8% weakly for CA-III. It is suggested that some fibers of types IIA and IIB contained CA-III. Further, Barnard *et al*. [17] noted that muscular dystrophy induced fiber composition changes in a characteristic manner. Fibers of types I, IIA, and IIB in the pectoral muscles from 50–80 day old DA chickens composed 0%, <2%–5%, and >90%–95% of the tissue. Pathological changes within the fibers occur selectively in the type IIB fibers. The minority type IIA fibers become slightly hypertrophied and after several weeks become pathologically rounded in DA chickens. The present study showed that the type I fibers show no pathological changes in the DA chickens, at least for several months.

In the present study, CA-III in the superficial pectoral muscle from DA chickens had a strong or weak immunohistochemical staining intensity at an equal frequency (21%). We suggest that some of the type IIB fibers in the DA chickens synthesized CA-III. The number of muscle fibers reacting strongly with anti-CA-III in the superficial pectoral muscles was about 12-times that in normal chickens. The muscle fibers stained with anti-CA-III in the superficial pectoral muscles of DA chickens were hypertrophied and became pathologically rounded with time. We, therefore, suggest that fiber types IIA and IIB contain CA-III in the superficial pectoral muscles of DA chickens.

The number of fibers in the tensor fasciae latae muscles and the obliquus externus abdominis muscles of the DA chickens staining strongly with anti-CA-III were about 3- and 2-times as numerous as in normal chickens, respectively. However, pathological changes within the fibers were not observed.

### Levels of CA-III and CA-II in plasma and erythrocytes

CA-III is relatively specific for skeletal muscle and should be useful as a diagnostic marker for various diseases [18,19]. Mokuno *et al.*[20] determined the serum CA-III levels in 143 patients with 4 types of progressive muscular dystrophy (limb-girdle dystrophy (LG), duchenne muscular dystrophy (DMD), facioscapulohumeral dystrophy (FSH), and congenital dystrophy). The serum CA-III levels in the LG, DMD, FSH, and congenital dystrophy patients were 0.8-, 2.5-, 0.3-, and 1.7-times that of normal individuals, respectively. In the present study, the plasma CA-III levels of 2 male muscular DA chickens were about 1.5-times that of the controls, as previously determined [13].

To our knowledge, no study on the CA-III and CA-II levels in the erythrocytes of human patients and chickens with muscular dystrophy has been published. In the present study, the CA-III levels in the erythrocytes of DA male chickens were about 15-times higher than those in normal male chickens, and 4-times higher than those in normal females [13]. As described above, Mizuno [7] and Park *et al*. [8] reported that SOD activities in the superficial pectoral muscle were significantly elevated in DA chickens. These findings suggest an increased turnover of reactive oxygen species such as superoxide anion or hydrogen peroxide in cases of muscular dystrophy.

Georgieva *et al*. [21] suggested that the compensatory elevation of erythrocyte catalase levels in chickens with alimentary muscular dystrophy was due to impaired antioxidant status following the oxidative and ecological stress produced by the disease.

In the present study, elevation of the CA-III level in the erythrocytes of DA chickens was probably induced by an increase in reactive oxygen species. However, to confirm this, determination of CA-III levels in the plasma and erythrocytes of DA chickens will be required. Nevertheless, our results suggest that chicken CA-III is a likely biomarker of radical-induced damage.

The erythrocyte CA-II levels in male DA chickens were similar to those in normal male and females [14]. These data suggest that synthesis of CA-II in chicken erythrocytes is not affected by reactive oxygen species.

## Conclusions

The levels of CA-III in the superficial pectoral, tensor fasciae latae muscles, and the plasma and erythrocytes from chickens with muscular dystrophy were higher than those in the normal chickens. The increase of CA-III levels in the muscles was visualized by CA-III immunohistochemistry showing an increase in both strongly and weakly stained fibers. These results demonstrate that CA-III is an effective marker for muscular dystrophy in the chicken; this finding also has potential for applications in other animals and in humans.

## Competing interests

The authors declare that they have no competing interests.

## Authors’ contributions

TN carried out the study design and performed the data analyses and also made the draft of the manuscript. DY and NI collected samples, processing and carried out the calculation of data. KO performed the data analyses and helped to draft the manuscript. KA contributed to the study design, the analyses of data and the writing of the manuscript. All authors read and approved the final manuscript.
